# Abnormal thalamocortical connectivity of preterm infants with elevated thyroid stimulating hormone identified with diffusion tensor imaging

**DOI:** 10.1038/s41598-022-12864-4

**Published:** 2022-06-03

**Authors:** Yong Hun Jang, Jinsup Kim, Sangwoo Kim, Kyungmi Lee, Jae Yoon Na, Ja-Hye Ahn, Hyuna Kim, Bung-Nyun Kim, Hyun Ju Lee

**Affiliations:** 1 Department of Translational Medicine, Hanyang University Graduate School of Biomedical Science and Engineering, Seoul, Republic of Korea; 2 Department of Pediatrics, Hanyang University Hospital, Hanyang University College of Medicine, Seoul, Republic of Korea; 3 Department of Radiological Science, Daewon University College, Jecheon, Republic of Korea; 4 Department of Child Psychotherapy, Hanyang University Graduate School of Medicine, Seoul, Republic of Korea; 5 Clinical Research Institute of Developmental Medicine, Seoul Hanyang University Hospital, Seoul, Republic of Korea; 6 Division of Child and Adolescent Psychiatry, Department of Psychiatry and Institute of Human Behavioral Medicine, Seoul National University College of Medicine, Seoul, Republic of Korea

**Keywords:** Paediatric research, Endocrinology

## Abstract

While thyroid disturbances during perinatal and postnatal periods in preterm infants with congenital hypothyroidism reportedly disrupt neuronal development, no study has considered the effect of thyroid disturbances in premature infants with subclinical hypothyroidism with elevations of thyroid stimulating hormone. We aimed to identify altered fiber integrity from the thalamus to cortices in preterm infants with subclinical hypothyroidism. All preterm infants born were categorized according to thyroid stimulating hormone levels through serial thyroid function tests (36 preterm controls and 29 preterm infants with subclinical hypothyroidism). Diffusion tensor images were acquired to determine differences in thalamocortical fiber lengths between the groups, and cerebral asymmetries were investigated to observe neurodevelopmental changes. Thalamocortical fiber lengths in the subclinical hypothyroidism group were significantly reduced in the bilateral superior temporal gyrus, heschl’s gyrus, lingual gyrus, and calcarine cortex (all *p* < 0.05). According to the asymmetric value in the orbitofrontal regions, there is a left dominance in the subclinical hypothyroidism group contrary to the controls (*p* = 0.012), and that of the cuneus areas showed significant decreases in the subclinical hypothyroidism group (*p* = 0.035). These findings could reflect altered neurodevelopment, which could help treatment plans using biomarkers for subclinical hypothyroidism.

## Introduction

Thyroid hormones (TH) play an essential role in human growth and neurodevelopment from the fetus to the first 2–3 years of life^1^. Thyroid dysfunction in preterm infants is associated with the immaturity of the hypothalamic-pituitary-thyroid axis, impaired synthesis and metabolism of TH, increased demand for thyroid hormone due to nonthyroidal illness, and drug administration^2^. One such disorder related to thyroid dysfunction, congenital hypothyroidism (CH), was a common etiology of severe cognitive impairment among preterm infants^2,3^. The clinical spectrum of CH spans from developmental delay to various neurologic problems, such as squint and functional impairment of hearing, speech, and voluntary motor activity. Screening for CH is performed using dry blood spot thyroid stimulating hormone (TSH) and thyroxine (T4) in the first week after birth^3,4^. However, the incidence of the delayed elevation of TSH levels that was missed in the first screening is high among very low birth weight (VLBW) and extremely low birth weight (ELBW) infants with CH^5–8^. As preterm or LBW and VLBW infants are also at high risk of developing cognitive dysfunction, repeated screening is strongly recommended^3^.

Although elevated TSH levels in preterm infants are detected through consecutive screening programs, the TSH cut-off value for neonatal screening has another important reason to prevent neurodevelopmental disorders and optimize developmental outcomes. The decision to start treatment is based on the results of venous thyroid function test, TSH levels of above 20 mU/L, and/or low free thyroxine concentration^3^. When TSH levels are between 6 and 20 mU/L with normal free thyroxine (fT4), management decisions become controversial; however, if TSH levels remain high for more than 3–6 weeks between preterm birth and term-equivalent age (TEA), many clinicians recommend levothyroxine supplementation^3,9,10^. Although the neurodevelopmental outcome in compensated or relatively mild cases of subclinical hypothyroidism (SH, normal free T4 values associated with moderate TSH elevation) is unclear, hypothyroidism is detrimental to neurodevelopment and can lead to developmental delay and cognitive dysfunction^11–14^. While CH can cause developmental disorders that clearly affect bone density, body weight, and cognitive function^11,15^, SH takes years to induce apparent physical symptoms^16^; hence, early thyroid symptoms indicative of SH are difficult to diagnose in a few weeks due to the absence of remarkable clinical symptoms^10^. Thus, reasonable clinical markers of SH in preterm newborns are needed to ensure the integrity of their neurodevelopment.

Thalamocortical tracts grow rapidly from 25 weeks of gestational age to TEA to become major white matter organizations that contribute to fundamental visual, auditory, motor, and somatosensory functions^17^. Its damage can cause disabilities of consciousness, working memory, sensory relay, and executive control throughout life^18^. TH plays important roles in neuronal migration, glial cell proliferation, synapse formation, axon and dendrite sprouting, and myelination: all essential for brain development during the first 2–3 years of life^1^. As TH are related to the activation of subplate neurons and participate in the mid and late phases of the maturation of oligodendrocytes by establishing transient synaptic contact from the thalamus to the cortices^19–22^, TH disturbances in perinatal and postnatal infants induce the reorganization and malfunction of cortical development^23^. Studies of rat models of early postnatal transient and chronic hypothyroidism have observed abnormal cellular integrity and reduced myelinated axons on electron microscopy, as well as increased T2-based relative signals on magnetic resonance imaging^23^; levothyroxine treatment has been found to be ineffective in protecting neuronal organizations^24,25^. Moreover, brain functional alterations in neurotransmitters, such as the ratio, density, and size of glutamate and inhibitory amino acids, have been found in the neocortex of rodents with gestational and early postnatal hypothyroidism^26^. Thus, we hypothesized that abnormal TH in preterm infants could affect thalamocortical connectivity due to impaired neuronal myelination or activation.

Diffusion tensor imaging (DTI) using magnetic resonance imaging (MRI) is a sensitive method for evaluating myelination abnormalities of white matter in the premature brain^27–33^. DTI studies of preterm infants have found that their thalamocortical connectivity is reduced compared to that of their term-equivalent counterparts^30^. The diminished connectivity was found to be positively correlated with cognitive dysfunction, as assessed through the Bayley-III (The Bayley Scales of Infant and Toddler Development, Third Edition), at the age of 2 years^34^. In addition, performance on the Bayley-II assessment of mental development and non-psychomotor outcomes is closely associated with the dysfunctional connectivity of the premature thalamus^35^. Although the degeneration of fiber integrity in premature brains has proven to be linked to developmental outcomes^34,35^, thyroid dysfunction commonly increases the risk of neurodevelopmental disabilities in VLBW infants^10,16^, making clinical treatments more ambiguous. Several studies regarding thyroid dysfunction have focused on morphometric analysis in children with congenital hypothyroidism^36^ or adults with hypothyroidism^37^, suggesting hypothyroidism can alter significantly white matter volume or cortical thickness. Moreover, a DTI study demonstrated an association between reduced white matter integrity in infants with CH and adverse cerebral abilities, including diminished hearing, nonverbal intelligence quotient, core language, and communication skills^38^. Another investigation using DTI showed high apparent diffusion coefficient values in the cerebral regions of premature infants with CH, which implies decreased cellular activity^39^. DTI can provide meaningful insight into SH-induced abnormalities in the fiber integrity of preterm infants, since SH are not only difficult to diagnose or treat in a few weeks because of the lack of remarkable clinical signs but are also connected to the advent of the CH^10,16^. However, whether these findings apply to preterm infants with SH remains unclear, and DTI investigations of the fiber connectivity of thalamocortical pathway in preterm infants with SH are needed. This study aimed to determine whether altered fiber integrity and asymmetries in thalamocortical pathways can distinguish preterm infants with SH from their healthy counterparts.

## Results

**Figure 1:**
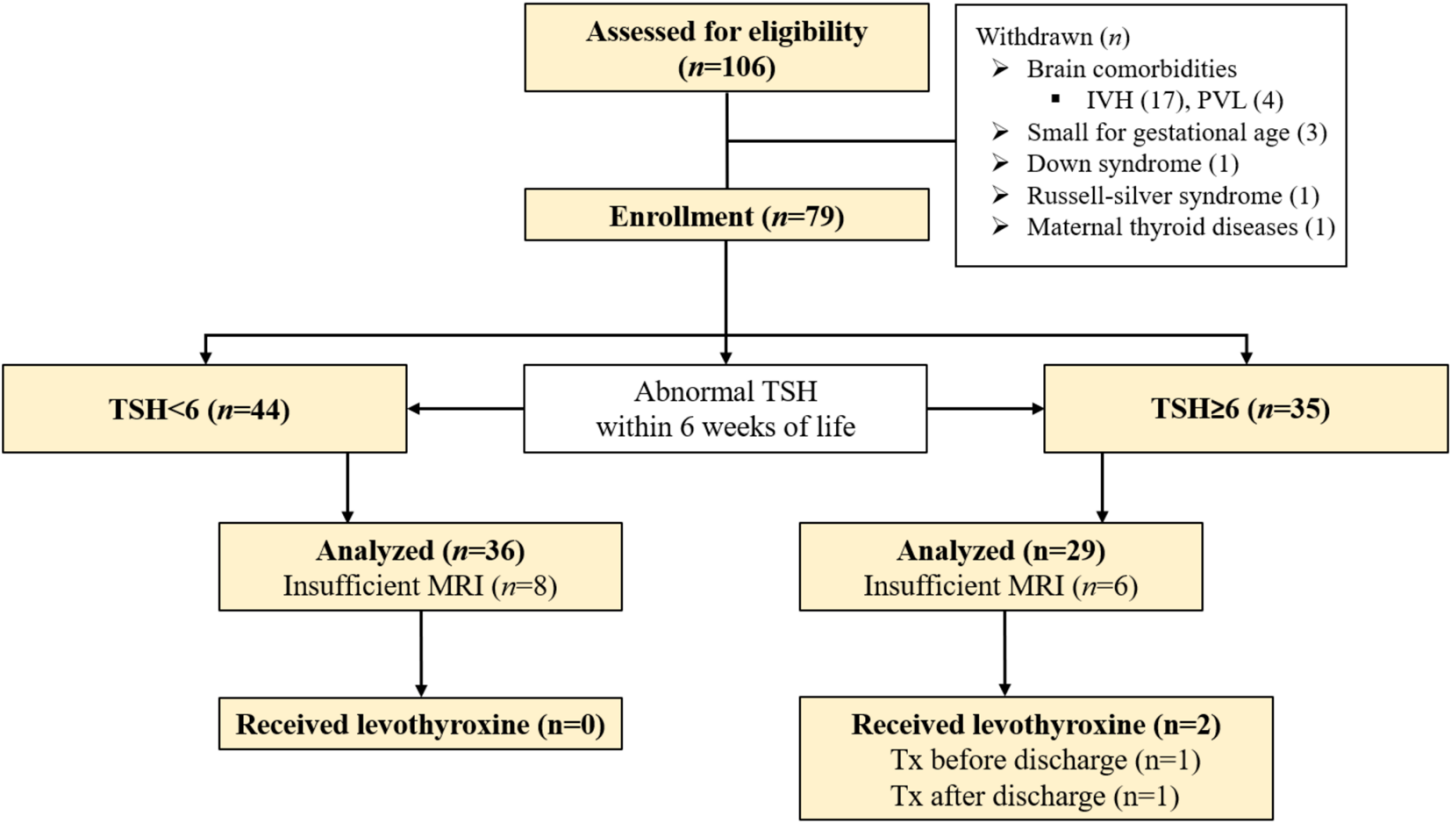
Schematic diagram of preterm infants with and without SH in the study. *SH* subclinical hypothyroidism, *IVH* interventricular haemorrhage, *PVL* periventricular leukomalacia, *TSH* thyroid stimulating hormone, *MRI* magnetic resonance imaging, *Tx* treatment.

We investigated a cohort of 106 preterm infants. After applying the exclusion criteria, a total of 27 infants were excluded: interventricular hemorrhage (n = 17), periventricular leukomalacia (n = 4), small for gestational age (n = 3), Down syndrome (n = 1), Russell-silver syndrome (n = 1), and maternal thyroid diseases (n = 1). In a cohort of 79 infants, we have classified infants with normal TSH levels (≥ 6; n = 35) and infants with abnormal TSH levels (< 6; n = 44) based on TSH levels within 6 weeks of life. Finally, after excluding 14 insufficient MR, the final cohort consisted of 29 preterm infants with SH and 36 preterm infants without SH (control group, CN) (Fig. 1). Additionally, 2 of SH group exhibited received levothyroxine (Tx before discharge, n = 1; Tx after discharge, n = 1). Among the 2 infants who were treated with levothyroxine, 1 infant born at 27 weeks’ gestation had normal results on the initial TSH and free T4 (fT4). Serum TSH levels gradually increased during follow-up, and at 6 weeks of life reached peak TSH levels (44 μU/mL) with normal fT4 levels (0.8 ng/mL). The other infant born at 26 weeks’ gestation was started on levothyroxine treatment after discharge with persistently elevated serum TSH levels (between 10 and 19.9 μU/mL). The TSH levels averaged across the thyroid function tests (TFT) were significantly higher in the SH group (11.56 ± 8.12 μU/mL) than in the CN (3.65 ± 1.37 μU/mL; *p* = 0.001). The individual TFT at each time point differed significantly between the SH group and CN group at the first (SH: 7.31 ± 1.07 μU/mL, CN: 1.74 ± 1.23 μU/mL, *p* < 0.001) and second time points (SH: 11.07 ± 7.70 μU/mL, CN: 3.29 ± 1.53 μU/mL, *p* < 0.001), but not at the third (SH: 8.39 ± 6.46 μU/mL, CN: 3.57 ± 1.55 μU/mL, *p* = 0.007) (Table 1). Furthermore, the individual fT4 levels were not significantly different from between the SH group and CN group with normal fT4 levels (> 0.7 ng/dL) at the first (SH: 1.63 ± 1.51 ng/mL, CN: 2.62 ± 1.98 ng/mL, *p* = 0.191), second (SH: 1.40 ± 0.32 ng/mL, CN: 1.34 ± 0.24 ng/mL, *p* = 0.374), and third (SH: 1.32 ± 0.19 ng/mL, CN: 1.40 ± 0.17 ng/mL, *p* = 0.230) time points. Gestational age, age at scan, birth weight, and body weight at MRI scan were not significant differences between the SH group and CN group (*p* > 0.05). Pre and postnatal treatment with steroid had no significant effect on the SH group and CN group (*p* > 0.05). The incidence of respiratory distress syndrome, necrotizing enterocolitis, and bronchopulmonary dysplasia were not significant differences in preterm infants with SH than in the controls (*p* > 0.05) (Table 1). Additionally, the 2 out of 29 infants with SH underwent surgical procedures of patent ductus arteriosus ligation and necrotizing enterocolitis operation with iodine exposure, and 1 infant underwent only patent ductus arteriosus ligation.

**Table 1:** Demographics and characteristics of preterm infants with and without SH.

Variables	CN (n = 36)	SH (n = 29)	*p-values*
TSH levels, μU/mL	3.65 ± 1.37	11.56 ± 8.12	0.001
Gestational age, weeks	29.36 ± 2.62	28.34 ± 2.17	0.099
Age at scan, weeks	37.83 ± 2.02	37.31 ± 1.85	0.286
Birth weight, kg	1.23 ± 0.26	1.12 ± 0.25	0.096
Body weight at MRI scan, kg	2.47 ± 0.61	2.44 ± 0.37	0.812
Apgar 1 min, score	3.69 ± 1.61	3.13 ± 1.48	0.157
Apgar 5 min, score	6.66 ± 0.92	6.31 ± 1.31	0.205
Cesarean section, n (%)	32 (88.9)	24 (82.8)	0.497
Patent ductus arteriosus, n (%)	20 (55.6)	18 (62.1)	0.623
Patent ductus arteriosus (requiring surgery), n (%)	6 (16.7)	3 (10.3)	0.720
Prenatal steroid, n (%)	21 (63.6)	20 (74.1)	0.419
Postnatal steroid, n (%)	6 (16.7)	7 (24.1)	0.540
Respiratory distress syndrome, n (%)	30 (83.3)	26 (89.7)	0.719
Culture-proven sepsis, n (%)	14 (38.9)	10 (34.5)	0.799
Necrotizing enterocolitis, n (%)	5 (13.9)	4 (13.8)	1.000
Necrotizing enterocolitis (requiring surgery), n (%)	4 (11.1)	2 (6.9)	0.684
Bronchopulmonary dysplasia, n (%)	19 (52.8)	16 (55.2)	1.000

All data are presented as averaged values with standard deviations or the number of counts in both groups.

*CN* controls, *SH* preterm infants with subclinical hypothyroidism, *TSH* thyroid stimulating hormone, *MRI* magnetic resonance imaging.

**Figure 2:**
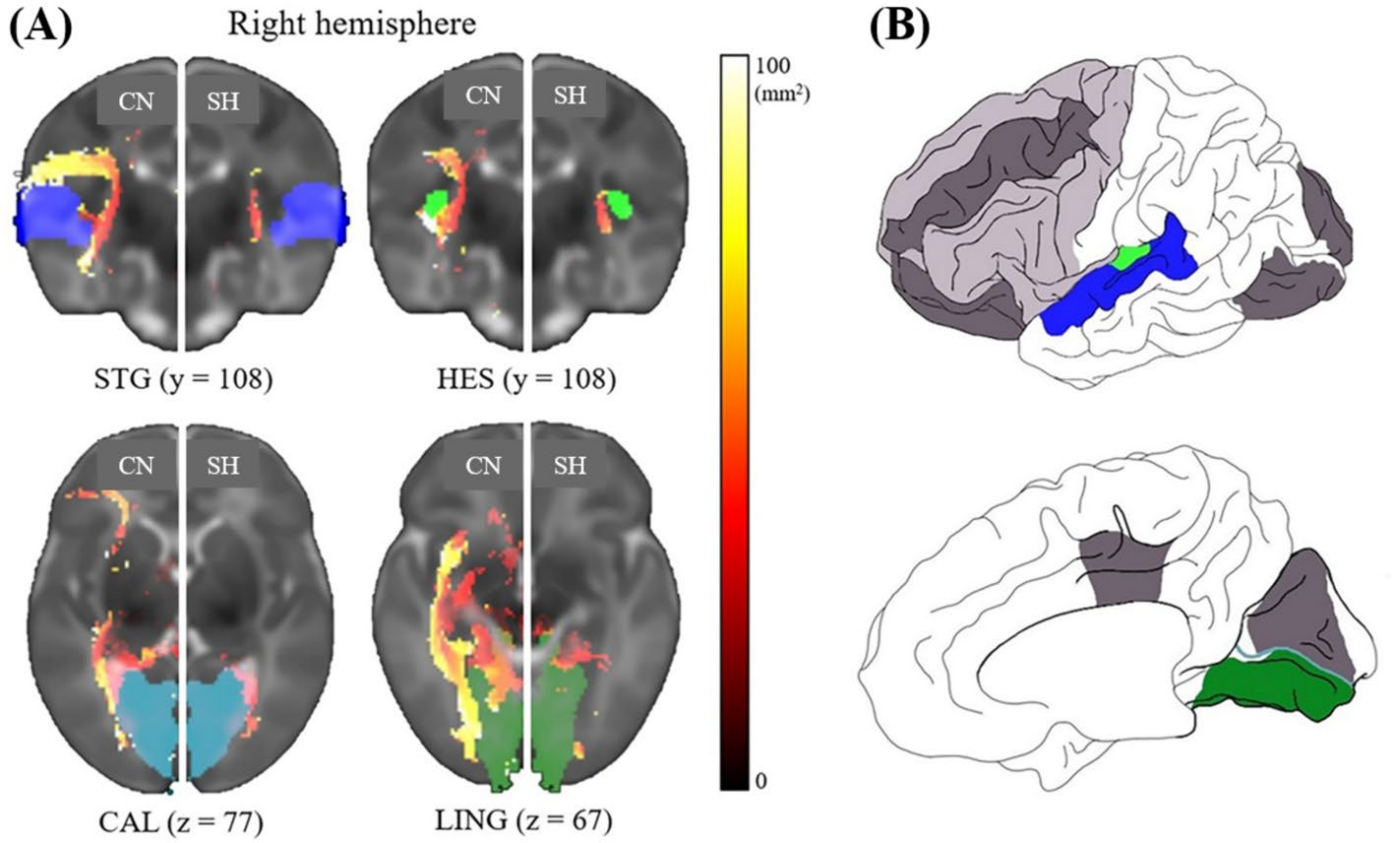
Differences in the fiber lengths between the controls and preterm with SH and a schematic illustration of the identified anatomical regions. (**A**) several areas with significant bilateral fiber reductions were identified (*p* < 0.05; mask regions = STG: blue, HES: green, CAL: sky blue, and LING: dark green). The gray template images are only present the right hemisphere, where that of the CN population was mirrored and placed against that of the SH group. The y and z indicate anterior–posterior and dorsal–ventral positions, respectively. (**B**) Schematics presented cerebral regions in which decreases in fiber lengths were observed. Dark gray indicates unilateral regions with significant fiber reductions, whereas the light gray indicates no meaningful regions in the UNC atlas. *CN* controls, *SH* preterm infants with subclinical hypothyroidism, *STG* superior temporal gyrus, *HES* heschl’s gyrus, *CAL* calcarine cortex, *LING* lingual gyrus.

**Figure 3:**
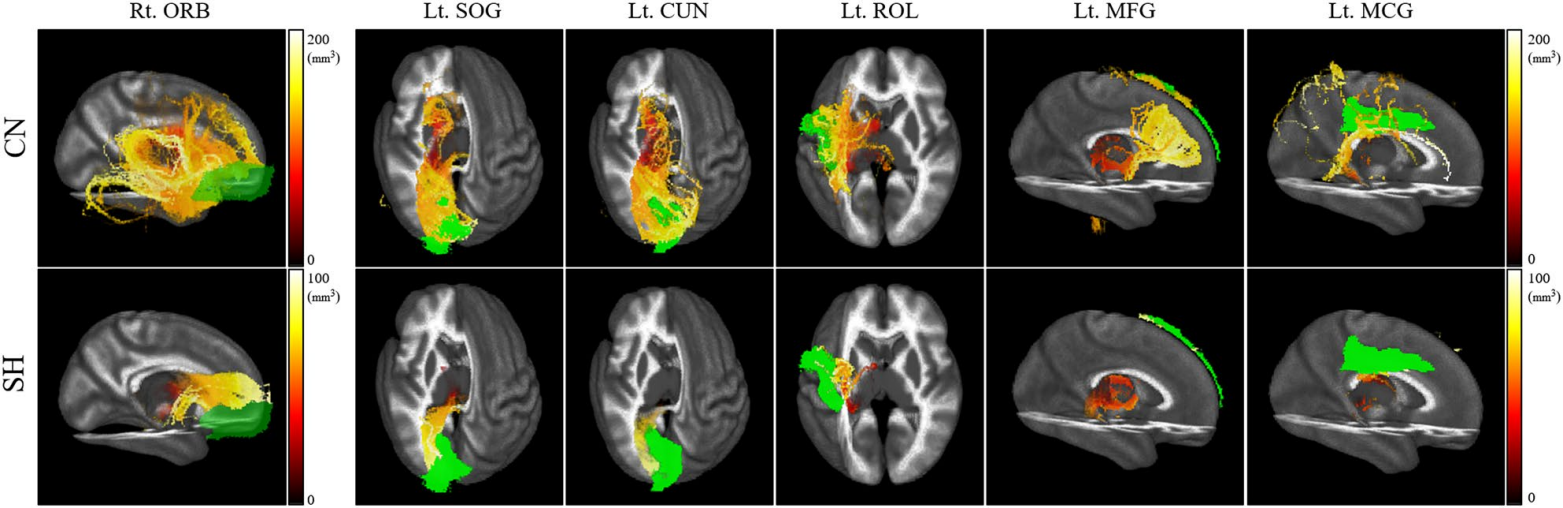
Comparison of unilateral fiber lengths between control and preterm infants with SH. The fiber lengths from the thalamus to each anatomical region (green color) significantly differed between the SH (bottom row) and control groups (upper row). *CN* controls, *SH* preterm infants with subclinical hypothyroidism, *ORB* orbitofrontal, *SOG* superior occipital gyrus, *CUN* cuneus, *ROL* rolandic operculum, *MFG* middle frontal gyrus, *MCG* middle cingulate gyrus.

The fiber lengths were measured to check the disturbances of the thalamocortical pathway in the SH. Compared to the healthy controls, infants with SH featured significant reductions in the fiber lengths in the cerebral regions of the SH. The bilateral abnormalities in fiber lengths included the superior temporal gyrus (STG), heschl’s gyrus (HES), calcarine cortex (CAL), and lingual gyrus. Abnormalities found only in the right and left hemispheres included the right orbitofrontal (ORB) and the left middle frontal gyrus (MFG), rolandic operculum (ROL), middle cingulate gyrus (MCG), superior occipital gyrus (SOG), and cuneus (CUN), respectively. In both cerebral regions, fiber lengths were 62–73% shorter in infants with SH relative to their CN counterparts (HES, 71.6%; CAL, 70%; LING, 68.7%; and STG, 63.5%; Fig. 2), accounting for the significant differences between the two populations (*p* < 0.05). Significant reductions in fiber lengths of 49.5 ~ 82.8% were observed in unilateral regions between the CN and SH groups: right ORB, 82.8%; the left SOG, 73.3%; left CUN, 68.2%; left MCG, 68.2%; left MFG, 65.7%; and left ROL, 49.5% (*p* < 0.05, Fig. 3). The fiber lengths are summarized in Table 2.

**Table 2:** Intergroup differences in the fiber lengths of the thalamocortical pathway.

Cerebral hemisphere	ROIs	Left hemisphere (mean ± SE)				Right hemisphere (mean ± SE)			
		CN	SH	t	*p-values*	CN	SH	t	*p-values*
**Bilateral**									
	STG	4.92 ± 0.53	1.63 ± 0.28	2.946	0.031	15.17 ± 1.82	5.27 ± 1.09	2.596	0.043
	HES	17.89 ± 2.52	5.29 ± 1.17	2.564	0.039	15.54 ± 1.94	4.16 ± 0.84	2.644	0.040
	CAL	22.87 ± 3.00	6.15 ± 0.98	2.525	0.043	22.22 ± 1.88	7.35 ± 1.08	2.555	0.041
	LING	21.09 ± 2.29	6.84 ± 0.90	2.554	0.039	20.58 ± 2.33	6.15 ± 0.98	2.852	0.044
**Unilateral**									
	MFG	28.42 ± 2.42	14.35 ± 1.52	4.143	0.048				
	ROL	15.08 ± 1.59	5.79 ± 0.90	3.015	0.041				
	MCG	18.48 ± 2.54	4.93 ± 1.07	2.683	0.041				
	SOG	25.27 ± 3.43	8.04 ± 1.55	2.691	0.043				
	CUN	18.26 ± 3.24	3.13 ± 0.79	2.941	0.041				
	ORB					24.16 ± 2.35	8.28 ± 1.96	3.838	0.043

The values are presented as the mean ± standard errors, adjusted for gestational weeks and age at scan as covariates.

*ROIs* regions of interest, *CN* controls, *SH* preterm infants with subclinical hypothyroidism, *SE* standard error, *STG* superior temporal gyrus, *HES* heschl’s gyrus, *CAL* calcarine cortex, *LING* lingual gyrus, *MFG* middle frontal gyrus, *ROL* rolandic operculum, *MCG* middle cingulate gyrus, *SOG* superior occipital gyrus, *CUN* cuneus, *ORB* orbitofrontal.

**Figure 4:**
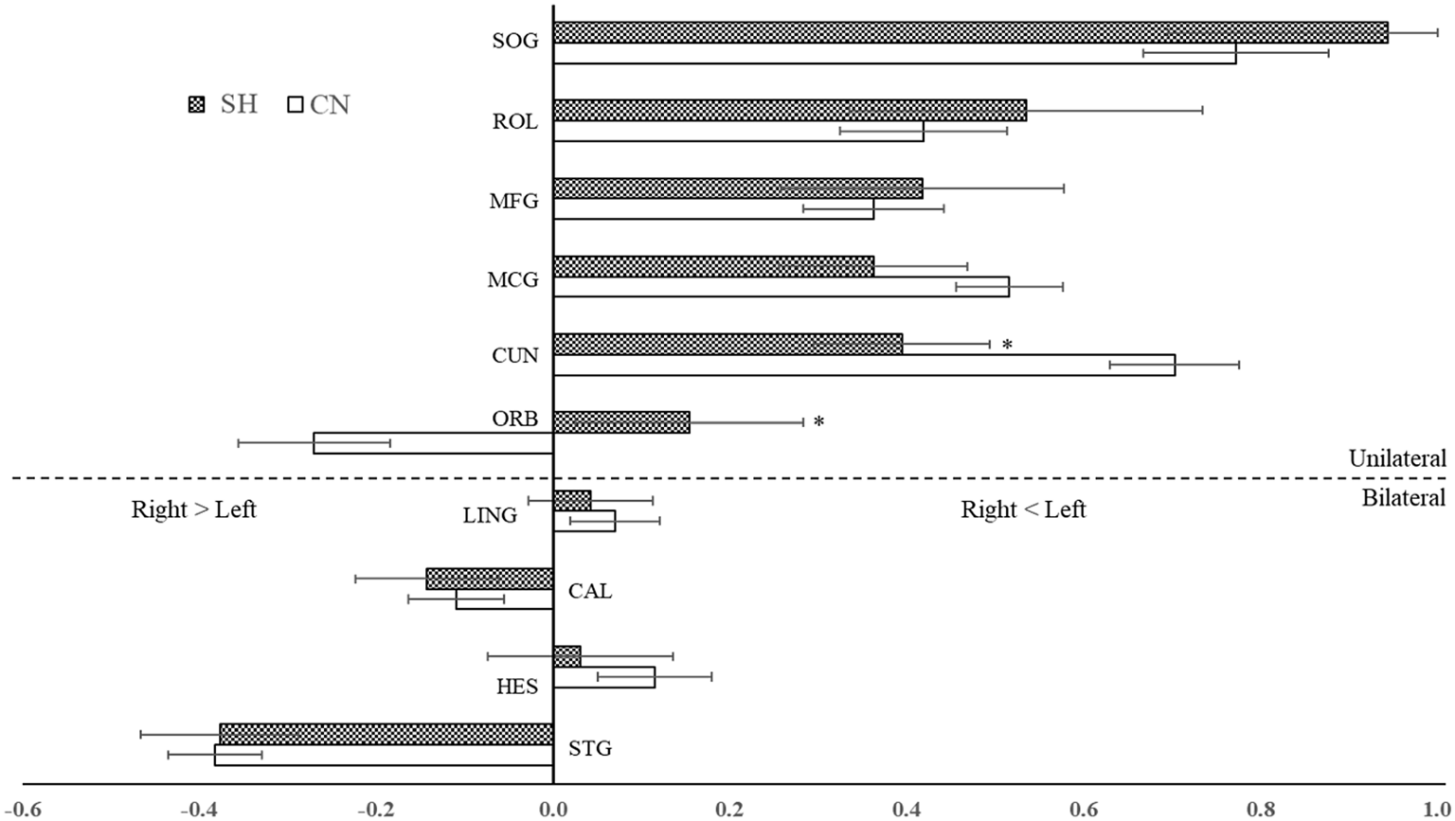
Changes in the lateralization indices of significant cerebral regions. The asymmetric values are altered according to two different patterns. The laterality of the ORB flipped between the SH and control, while the CUN exhibited a meaningful reduction (asterisks appear the regions of significant differences in the cerebral regions, *p* < 0.05). The dotted line demarcates bilateral from unilateral changes. *CN* controls, *SH* preterm infants with subclinical hypothyroidism, *STG* superior temporal gyrus, *HES* heschl’s gyrus, *CAL* calcarine cortex, *LING* lingual gyrus, *ORB* orbitofrontal, *SOG* superior occipital gyrus, *CUN* cuneus, *ROL* rolandic operculum, *MFG* middle frontal gyrus, *MCG* middle cingulate gyrus.

We also employed the lateralization index to assess hemispheric dominance in regions wherein the length of fiber tracts differed significantly between the SH and CN populations. The asymmetric values showed an overall left dominance, excepting the STG and CAL (Fig. 4). In the unilateral areas, while ORB was dominant in the right hemisphere of CN infants, it became left-dominant in the SH group (SH: 0.154 ± 0.129, CN: -0.270 ± 0.086, *p* = 0.012). In addition, the CUN was significantly lower in the SH group (0.395 ± 0.100) than in the CN group (0.703 ± 0.073; *p* = 0.035). The lateralization values in the other regions showed slight increases or decreases, but none were significant (Table 3).

**Table 3:** Mean lateralization indices in cerebral regions where the fiber lengths were significantly decreased in the bilateral and unilateral hemispheres.

Cerebral hemisphere	ROIs	Mean lateralization indices			
		CN	SH	F-test	*p-values*
**Bilateral**					
	STG	− 0.382 ± 0.053	− 0.378 ± 0.089	0.260	0.612
	HES	0.115 ± 0.064	0.032 ± 0.105	0.314	0.577
	CAL	− 0.108 ± 0.054	− 0.141 ± 0.081	0.257	0.614
	LING	0.070 ± 0.051	0.043 ± 0.071	0.005	0.942
**Unilateral**					
	Lt. CUN	0.703 ± 0.073	0.395 ± 0.100	4.650	0.035
	Lt. MCG	0.516 ± 0.060	0.362 ± 0.106	0.935	0.337
	Lt. MFG	0.363 ± 0.080	0.418 ± 0.160	0.095	0.759
	Lt. ROL	0.419 ± 0.094	0.535 ± 0.200	0.866	0.356
	Lt. SOG	0.772 ± 0.105	0.943 ± 0.248	1.014	0.318
	Rt. ORB	− 0.270 ± 0.086	0.154 ± 0.129	6.657	0.012

These values are presented as the mean with standard errors. The left CUN and the right ORB show significant differences between the CN and the SH groups.

*CN* controls, *SH* preterm infants with subclinical hypothyroidism, *ROIs* regions of interest, *STG* superior temporal gyrus, *HES* heschl’s gyrus, *CAL* calcarine cortex, *LING* lingual gyrus, *CUN* cuneus, *MCG* middle cingulate gyrus, *MFG* middle frontal gyrus, *ROL* rolandic operculum, *SOG* superior occipital gyrus, *ORB* orbitofrontal, *Lt* left, *Rt* right.

## Discussion

In this study, we employed DTI to quantify the fiber integrity of preterm infants with SH and thus determined the significant reductions in fiber lengths in the thalamocortical pathways of infants with SH in the following regions: the bilateral STG, HES, CAL, and LING; and the unilateral ORB, MCG, MFG, ROL, SOG, and CUN. In addition, compared to controls, the lateralization values of two cerebral regions significantly changed; the laterality of the ORB was transposed to the opposite hemisphere in infants with SH, and that of the CUN decreased significantly. These results indicate disrupted fiber connectivity of the thalamocortical pathway in the frontal, temporal, and occipital lobes of infants with SH, reflecting abnormal white matter myelination and impaired cortical growth in this clinical population.

It is noteworthy that the fiber lengths in the SH group were significantly decreased in the temporal lobe, including the regions of the STG and HES, suggesting reductions of dendrite and synaptic extensions. TH regulate the generation and number of oligodendrocytes, which are related to the activation of subplate neurons that help to establish transient synaptic contact from the thalamus to the cortex^40–42^. The development of subplate neurons is prominent at mid- and late-fetal phases, and disturbances during these periods induce the reorganization of the cortical development in perinatal and early postnatal infants^43^. Several studies on CH have found cortical thinning in the temporal lobe of children and adults with regard to neuronal loss in the temporal lobe^36,37^. In rodent models of transient and CH, abnormal cellular integrity has also been observed using electron microscopy^24,25^, implicating TH in neuronal death. Moreover, adult-human connectome research has reported that short fibers are positively correlated with lower cortical thickness^44^, which supports our hypothesis that the reduced fiber lengths of preterm infants suffering from SH could be used as a biomarker to signal neuronal deficits in the temporal lobe, since the advent of the SH precedes thyroid dysfunction in preterm infants^7^. DTI could be a more sensitive marker for monitoring neurodevelopment than the morphometric analyses based on three-dimensional T1-weighted imaging^45^. However, further studies are needed to validate the relationship between fiber length and cortical thinning in preterm infants.

We also demonstrated significant bilateral fiber reductions in the occipital and frontal lobes (CAL and LING, Fig. 2) and the lobes that exhibited unilateral changes in fiber length (Fig. 3), and the lateralization states of the CUN and ORB in the frontal and occipital lobes, respectively, differed clearly between the two populations (Fig. 4). These phenomena could be expected to postpone normal neonate growth, causing abnormal neurodevelopment patterns. In the fetal stages, the thalamocortical pathway first progresses to the anterior and middle cerebral regions until 21 weeks of gestational age, matures posteriorly from 22 weeks of gestational age, and condenses and organizes the projection of specific cerebral cortex until 40 weeks of gestational age^17^. Interestingly, similar to our findings, a DTI study of infants born before 28 weeks with low T4 concentrations observed increased apparent diffusion coefficient values in the occipital region at term-equivalent ages, indicating poor white matter formation due to thyroid disturbance^39,45^. This finding further suggests that, in the context of the thyroid hormone-activated sensitive period of mid- and late- fetal development^43^, the abnormal thyroid state affects that the decreased fiber lengths in the SH could indicate the postponement of fiber aggregation in the occipital lobe. On the other hand, there are developmental differences in cerebral volume, sulci, and fiber connectivity in fetuses, preterm infants and full-term neonates^46–48^. The ORB volumes in healthy fetuses^48^, the lateral ORB network in full-term neonates^46^, and the frontal sulci in preterm infants^47^ favor right hemispheric dominance across aging. In addition, the centrality of the CUN shows left dominance in neonates^46^, which is comparable to the lateralization features of preterm controls in this study. However, our preterm infants with the SH exhibited a different pattern of asymmetries in the CUN and ORB, compared to preterm controls (Fig. 4). This result indicates that the changed lateralization in the occipital and frontal lobes may represent neurodevelopmental disorders caused by defective fiber maturation. Consequently, we suggest that the decreased fiber lengths in the corresponding cerebral regions accompanying the lateralization changes would manifest as developmental delay due to the slow activation of the thyroid hormone. However, additional studies using DTI are required to investigate the longitudinal lateralization patterns of preterm infants with SH, as increased cortical thickness in the occipital and frontal regions in infants with the CH has been implicated in developmental deformation due to the lengthened maturation in adolescence consequent of CH^36^.

Most neuroimaging studies related to CH have identified abnormalities in various cerebral regions, including the frontal, temporal, and occipital lobes^36,37,39^, resulting in memory and visuospatial disorders in relation to cognitive function at school ages^36,49–51^. These symptoms have also been found in children with constant thyroid disturbance during the neonatal stage^52^, which could be perceived as a valuable clinical warning for preterm infants with thyroid dysfunction. Importantly, the thyroid hormone should be more carefully monitored in the early birth, since 17–40% of newborns with abnormal TH levels develop CH^10^. However, thyroid symptoms are difficult to diagnose within a few weeks of birth because of the lack of remarkable clinical signs during this period^53^. We found a reduction in fiber length in the SH as a prodrome of thyroid pathophysiology using a non-invasive DTI approach, implying that it can be used as a reliable imaging biomarker to assist clinically efficient cures for the normal development of preterm infants. Furthermore, fiber abnormalities identified using DTI have been positively correlated with mental, cognitive, and motor function in preterm infants^35,54^. When combined with serial thyroid testing, fiber changes determined with DTI could be used as a quantitative clinical marker to help anticipate whether cerebral development appropriately progresses while preterm infants with SH undergo treatment.

This study is subject to several limitations that need to be addressed in future research. First, this study employed a single-center, retrospective, and uncontrolled observational design, and was additionally limited by variations in the timing and interval of TFT screening. Moreover, we did not account for other potential confounders, including maternal factors of iodine intake, drug use, the existence of thyroid disease or thyroid autoantibodies, and those that depended entirely on the decisions of the attending pediatric endocrinologists, including variations in the indication, timing, and duration of levothyroxine replacement therapy. We also note that the small number of infants subjected to MRI and DTI analyses was insufficient for the subgroup analysis of SH and transient hypothyroxinemia of prematurity. Furthermore, we did not monitor iodine exposure in infants, although we routinely used 0.5% chlorhexidine instead of iodine-containing agents as skin antiseptics during the procedures at our institute throughout this study period. As only 10% of patients underwent surgical procedures, the effect of iodine exposure on the incidence of TSH elevation might be insignificant. Regarding imaging analysis, recent DTI studies have been implemented with multi-shell imaging techniques with two or more different b-values to show fiber distributions of the white matter more clearly^55^. However, as the DTI dataset was acquired with a b-value of 1000 s/mm^2^ and the number of 16 directions. These could cause a different sensitivity of the fiber lengths due to a variety of diffusion factors such as diffusion anisotropy, crossing fibers, and the number of axons in a voxel^56^, which can reduce the quality of tract reconstruction. Additional studies with high b-value and gradient directions can reinforce the outcomes of this study in the future. In addition, thalamic volume can affect the potential difference of fiber lengths of thalamocortical tract. The relationship between structure alteration and fiber integrity should be further explored by follow-up studies to consolidate the present study.

We investigated fiber lengths using DTI to identify abnormalities in the thalamocortical pathway in preterm infants with SH. Defective fiber integrity accompanied by lateralization changes were demonstrated in several cerebral regions, including the frontal, temporal, and occipital lobes, of infants with SH. These results could help to establish a reasonable plan for the treatment of thyroid dysfunction and thus prevent the onset of cognitive impairments related to memory and visuospatial disorders in toddlers, children, and adults.

## Methods

### Study populations

All preterm infants who were admitted to the neonatal intensive care unit of Hanyang University Clinic for Developmental Disorders were initially recruited to investigate their cerebral development between February 2016 and January 2019. Preterm birth was defined as being born before 32 weeks of gestation with a birth weight of < 1.5 kg. All recruited infants were confirmed through MRI and ultrasonography screenings to have no brain injuries at near-term ages (postmenstrual age, 36–41 weeks), such as focal abnormalities, intraventricular hemorrhage, chromosomal abnormalities, congenital infections, cystic periventricular leukomalacia, and Down syndrome. Infants with SH were distinguished from those without SH by TSH values of > 6 and < 20 μU/mL; the measurements were averaged across three serial TFT administered at 1, 3, and 6 weeks of life. Clinical characteristics included gestational age, age at scan, birth weight, body weight at MRI scan, Apgar scores at 1 and 5 min, cesarean section, patent ductus arteriosus and it with requiring surgery, prenatal and postnatal steroid, respiratory distress syndrome, culture-proven sepsis, necrotizing enterocolitis and it with requiring surgery, and bronchopulmonary dysplasia. Serial TFTs measuring both serum TSH and fT4 levels were routinely performed at 1, 3, and 6 weeks of postnatal age for all VLBW infants. Even if the initial TFT values obtained at 7 days of life were normal, we repeated the screening test at 3 weeks (from 14 to 21 days) and 6 weeks (from 42 to 49 days of life) because delayed elevations of TSH elevation is common among premature infants^6^. If the TFT values were abnormal (TSH > 6 μU/mL and/or fT4 < 0.8 ng/dL), the test was repeated after 1 or 2 weeks according to careful consideration by the pediatric endocrinologists at our hospital. The TSH levels measured through serial TFTs were calculated as average values with standard deviations for each and total steps. The Hanyang University Hospital Institutional Review Board (IRB No. 202101015) approved this study protocol, and informed consent for participation in this study was acquired by the parents. All procedures were performed in compliance with the principles of the Declaration of Helsinki.

### MRI acquisitions

The CN and SH groups were examined at near-term ages (PMA 36–41 weeks) using whole-body 3 T MRI (Philips, Achieva, Best, Netherlands) with a 16-channel phase-array head coil during natural sleep while they were covered with a blanket to maintain body temperature. Their safety was ensured by monitoring pulse oximetry, respiratory rate, and heart rate by a trained pediatrician during the MR scans. For the DTI, three-dimensional (3D) echo-planar images with a single-shot spin-echo type were acquired with volume B0-shimming. The scan parameters of the DTI were as follows: TR = 4800 ms, TE = 75 ms, field of view (FOV) = 120 × 120 mm^2^, voxel sizes = 1.56 × 1.56 mm^2^, slice thickness = 2 mm, slice numbers = 50, flip angle = 90 degrees, water-fat shift = 4.68 Hz/pixel, number of average = 2, directions = 16, b-values = 1000, total acquisition time = 6 min 17 s. In addition, structural T2-weighted images were obtained to exclude preterm infants with white matter abnormalities. The parameters for the scan were as follows: TR = 4800 ms, TE = 90 ms, FOV = 180 × 180 mm^2^, voxel sizes = 0.5 × 0.5 mm^2^, slice thickness = 3 mm, slice numbers = 25, flip angle = 90 degrees, water-fat shift = 4.68 Hz/pixel, number of average = 1, total acquisition time = 6 min 30 s.

### Reconstruction and quantification for fiber connectivity

**Figure 5:**
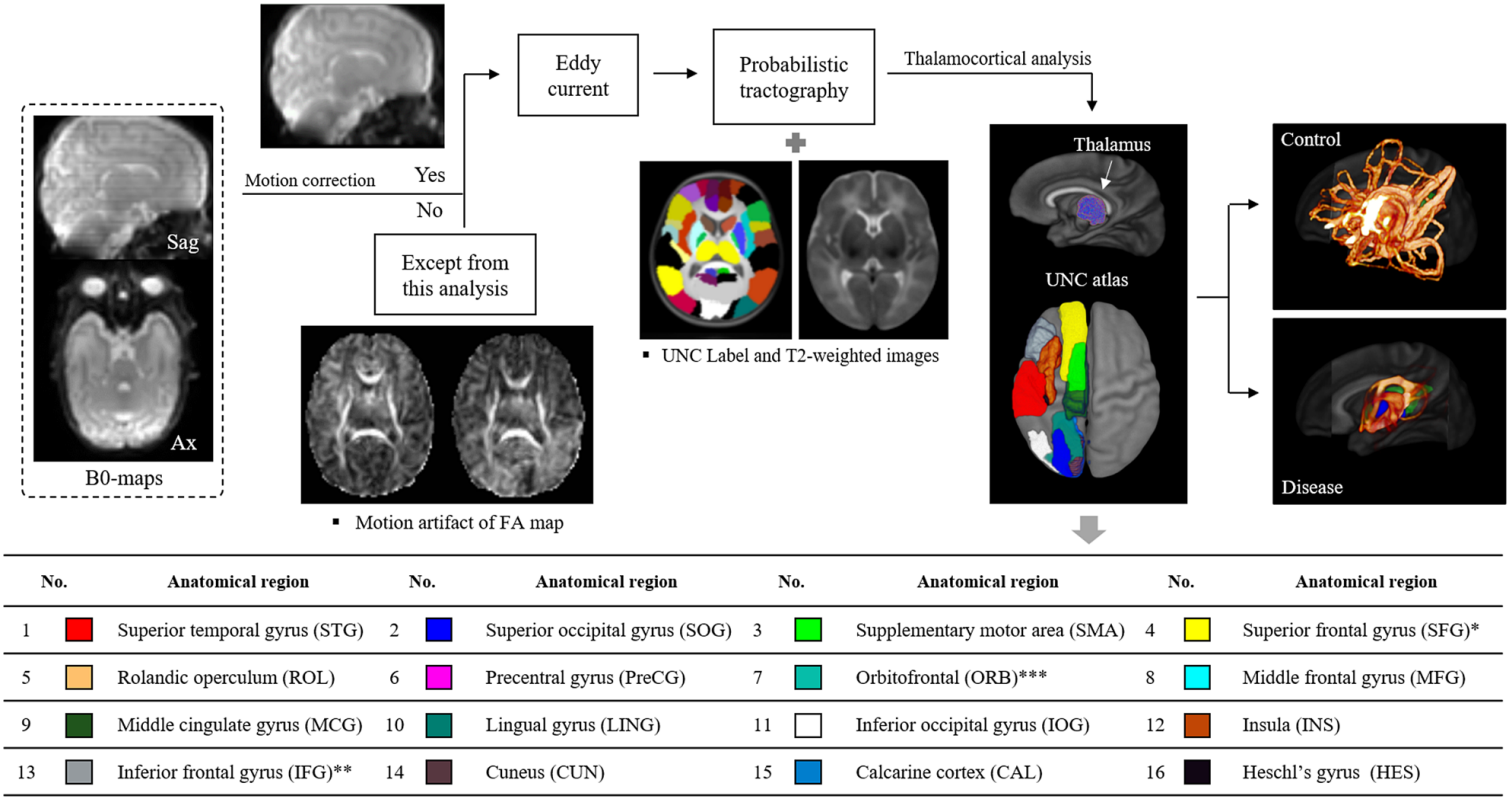
An illustration of the extraction of tractography-based fiber lengths. Image datasets were chosen according to whether motion artifacts were corrected. After eddy-current correction, the probabilistic process was initiated to reconstruct fiber lengths. The seed point was set to the thalamus, and each endpoint is present in the UNC label-map (colorful regions overlaid on a gray morphology atlas). The color-map regions used to mask the image are marked in a table with each anatomical name. The number of asterisks appears the combination of several anatomical regions, which include that * is dorsal and medial superior frontal gyrus, ** is pars triangularis and pars opercularis, and *** is superior, middle, inferior, and medial orbitofrontal gyrus. *Sag* sagittal, *Ax* axial, *UNC* University of North Carolina atlas.

The 3D-DTI images were used to reconstruct the thalamocortical connectivity based on fiber length using the FMRIB Software Library (FSL, https://fsl.fmrib.ox.ac.uk/fsl/fslwiki)^57^. First, we implemented motion correction using mcflirt, which is a part of the FSL^58^. Twenty preterm infants whose fractional anisotropy maps showed motion artifacts were removed from the DTI analysis (Fig. 5); hence, we were able to determine fiber integrity for 65 infants (CN, 39; SH, 29). Second, an early eddy-current technique provided in the FSL (eddy_correct) was applied to the DTI images to correct subject distortions and movements^59^. We obtained a parenchymal b0-map, processed it using the Brain Extraction Tool (BET)^60^ from 16 directional DTI images, applied linear registration from diffusion space to a neonatal T2-weighted image of the University of North Carolina (UNC) atlases^61^, and vice versa. Subsequently, the bedpostx GPU-version^62^, which default corrects partial volume effect^63^ since the slice thickness of this study was a bit thick, was executed with the advanced options (Fibers 3 and Rician option for uniform noise levels) to rapidly estimate fiber distributions for each voxel of the brain. The GPU-based probtrackx was also employed to extract information related to fiber connectivity between the thalamus and various cerebral regions^64^. The thalamus in the neonatal UNC-labels was assigned as the main seed, and each cerebral UNC region was allotted as a stop criterion to check the left and right thalamocortical integrity. The regions of interest from the thalamus to each cortex were defined by Ball et al.^30^, as illustrated in Fig. 5. Lastly, the threshold-free cluster enhancement (TFCE)^65^ of the FSL was determined with randomize to find the significant thalamocortical tracts between the control and the SH. The parameters for the TFCE were designed for a two-sample unpaired t-test without not modeling mean values in the design matrix. Subsequently, the threshold value was selected with 0.95; it is equivalent to *p* < 0.05. We controlled family-wise error rate after TFCE.

### Fiber lateralization of the thalamocortical tracts

Cerebral myelination in term-born neonates progresses rapidly in the whole brain between 6–20 months; between 21–48 months of life, myelination gradually escalates in the frontal and temporal lobes relative to the parietal and occipital lobes^66^. In addition, the cerebral regions of newborns feature neurodevelopmental asymmetries. Some regions, such as the superior temporal gyrus and orbitofrontal gyrus, develop independently to become dominant in the left or right hemisphere^46–48^. This extent of this difference is determined by lateralization evaluations^46^. Thus, we utilized the lateralization index and TFCE analysis to check whether the developmental processes of the significant thalamocortical tracts were changed:

$$ {\text{LI}} = \frac{{{\text{Lt}} - {\text{Rt}}}}{{\left( {{\text{Lt}} + {\text{Rt}}} \right)}}{ } $$

where Lt is the left-dependent fiber length, and Rt is the right. Positive LI values indicate left-dominant function, while negative values indicate right dominance. Lastly, each LI between the CN and the SH was averaged with standard errors and then compared to investigate the developmental asymmetries of infants with SH with their CN counterparts.

### Statistical analysis for subjects and lateralization indices

Although the significant thalamocortical tracts of the fiber lengths were extracted through the TFCE in the FSL, the differences in subject populations and cerebral asymmetries were evaluated using SPSS 22.0. The statistical analysis was conducted with the 5000 permutations and two-group differences. The analysis was adjusted for the covariates of gestational weeks and age at scan. The corresponding values for the significant UNC tracts were extracted using the cluster function of the FSL, which was calculated as average values with standard errors. The chi-squared distributions and independent t-tests were implemented for demographic and clinical characteristics. We also analyzed each LI value of the significant thalamocortical regions calculated with fiber lengths, which were evaluated by ANCOVA adjusting the gestational age and the age at scan. The comparison of the main effect was conducted with Bonferroni correction, and the adjusted *p* value was assigned to a significant range of < 0.05.

## Data Availability

The datasets generated during and/or analyzed during the current study are not publicly available due to inability to share personal information according to research ethics but are available from the corresponding author on reasonable request. Correspondence and requests for materials should be addressed to Y.H.J (ryanjang93@hanyang.ac.kr) or H.J.L (blesslee77@hanmail.net).
